# Unpacking Nutrition Integration in Irish Healthcare: A Qualitative Exploration Across Five Clinical Disciplines

**DOI:** 10.1111/nbu.70058

**Published:** 2026-05-31

**Authors:** Sarah O'Donovan, Aoife Gillane, Sarah Scully, Gemma McMonagle, Laura Keaver, Rónán Doherty, Lisa Ryan

**Affiliations:** ^1^ Department of Sport, Exercise and Nutrition Atlantic Technological University Galway Ireland; ^2^ Department of Health and Nutritional Science Atlantic Technological University Sligo Ireland; ^3^ Department of Tourism and Sport Atlantic Technological University Letterkenny Ireland

**Keywords:** education reform, healthcare professionals, Irish healthcare, nutrition education, nutrition in healthcare, scope of practice

## Abstract

Nutrition care is fundamental to effective healthcare; nevertheless, its everyday implementation by Irish healthcare professionals (HCPs) is poorly understood. This study aimed to explore the knowledge, practice, and perceived barriers to delivering nutrition care among HCPs and students in medicine, nursing, pharmacy, physiotherapy and occupational therapy in Ireland. A qualitative design using semi‐structured interviews was conducted with 37 purposively selected HCPs and students from diverse clinical and primary care settings. Data were analysed using reflexive thematic analysis to identify key patterns and insights. Three central themes were identified: (1) Professional Boundaries, Confidence & Perceived Competence in Nutrition Knowledge, (2) Structural & Logistical Barriers to Nutrition Integration and (3) Variability in Nutrition Integration & Its Impact on Healthcare Practice. Participants universally acknowledged the importance of nutrition; however, unclear role boundaries, limited formal education, inconsistent referral processes and resource constraints hindered its implementation. Confidence in providing nutrition advice was largely driven by personal initiative rather than structured education, leading to inconsistent care. Uncertainty surrounding referral pathways, and concerns about burdening colleagues compounded barriers to effective nutrition support. Despite valuing interdisciplinary collaboration, siloed working practices resulted in missed opportunities for timely, coordinated nutrition interventions—especially for patients with complex needs. A disconnect exists between the acknowledged value of nutrition care and its practical integration into Irish healthcare. Addressing this gap requires enhanced undergraduate and postgraduate training, clearer referral frameworks, stronger interprofessional collaboration and improved access to dietetic support. These measures are essential to strengthen nutrition care delivery and improve patient outcomes across healthcare settings.

## 1 Introduction

As diet‐related non‐communicable diseases (NCDs) continue to rise globally, alongside the concurrent challenges of obesity and malnutrition, it is imperative that HCPs are equipped with fundamental, evidence‐based nutrition knowledge to optimise healthcare outcomes (Adamski et al. 2018). Poor dietary habits are among the leading causes of morbidity and mortality worldwide, with the 2017 Global Burden of Disease study identifying unhealthy diets as a key contributor to preventable deaths (Adamski et al. 2018; Tan et al. 2024; Shan et al. 2023; GBD 2023 IHD and Dietary Risk Factors Collaborators 2026). Research suggests that up to 80% of chronic illnesses and premature mortality can be mitigated through modifiable lifestyle factors, further highlighting the central role of nutrition in the prevention and management of chronic diseases such as diabetes, cardiovascular disease and hypertension (Katz et al. 2018; Carter et al. 2022; Ní Chonaill et al. 2023; Li et al. 2015). Despite its critical importance, nutrition remains inadequately integrated into healthcare education and practice, limiting HCPs' ability to provide effective nutrition care (Kris‐Etherton et al. 2014; Erickson et al. 2023; Keaver et al. 2018; Ball et al. 2010; McMonagle et al. 2023).

Nutrition care refers to the application of nutrition knowledge by HCPs to improve patient nutrition behaviours and promote health (Ball et al. 2010). Although dietitians are the primary nutrition experts across all clinical care settings, their limited availability requires other HCPs, such as doctors, nurses, physiotherapists, pharmacists and occupational therapists (OTs), to play an active role in identifying nutrition‐related issues and providing first‐line evidence‐based advice or making appropriate referrals (Griffin et al. 2024; Agwara et al. 2024; McClinchy et al. 2015; Peniamina and McLean 2024). These professionals, due to their regular patient interactions, are well‐positioned to provide essential first‐line dietary advice (Agwara et al. 2024; McClinchy et al. 2015; Ball et al. 2010, 2014; Aggarwal et al. 2020; Crowley et al. 2019). However, their ability to deliver effective care is constrained by inadequate training, role ambiguity, time constraints and scope of practice limitations (Adamski et al. 2018; Griffin et al. 2024; Agwara et al. 2024; McClinchy et al. 2015; Khiri and Howells 2025; Wong et al. 2025; Krishnan et al. 2024). Continuous professional development (CPD) and integration of first‐line dietary advice into pre‐registration training is essential to equip HCPs to integrate nutrition effectively into patient care (Agwara et al. 2024; Blunt and Kafatos 2019; Eyemienbai et al. 2025).

The gap in nutrition education is particularly evident in Ireland, where 37% of healthcare programmes have no reference to nutrition, and only 14% explicitly integrate it (McMonagle et al. 2023). Additionally, while two‐thirds of Irish physiotherapists encounter nutrition‐related issues in practice, only 37% have received formal nutrition training (Griffin et al. 2024). This is compounded by insufficient interdisciplinary coordination and the lack of standardised guidelines for integrating nutrition into healthcare curricula (Kris‐Etherton et al. 2014; McMonagle et al. 2023). Previous research indicates that non‐dietetic HCPs often manage malnutrition but lack the training and confidence to conduct effective screenings and interventions (Browne et al. 2022). Notably, Ireland is the only country among six developed nations without formal guidelines for integrating nutrition into medical education (Keaver et al. 2018; Crowley et al. 2015). These deficiencies undermine the ability of HCPs to address nutrition‐related issues, compromising patient outcomes and the efficacy of treatments, especially when patients are nutritionally depleted (Erickson et al. 2023). Addressing these gaps is vital to improving patient care and reducing the morbidity and mortality linked to nutrition‐related conditions (Ní Chonaill et al. 2023; Slawson et al. 2013).

A 2025 study explored the current state of nutrition care within primary healthcare in Ireland, identifying practices, barriers and potential interdisciplinary solutions (Eyemienbai et al. 2025). The study highlighted the crucial role of HCPs in delivering nutrition care, especially given the shortage of dietitians in the primary care setting, and revealed that while HCPs recognise the importance of nutrition, they face challenges such as limited time and inadequate training. The findings suggested a necessary shift toward an interdisciplinary care model that integrates nutrition services more effectively and provides enhanced training and resources for all primary care providers.

Building on these findings, this study addresses a critical gap in the literature by exploring the integration of nutrition into healthcare practice in Ireland, drawing from the perspectives of both HCPs and healthcare students. It investigates their knowledge, practices and perceptions, identifying key barriers and proposed enablers to embedding nutrition within healthcare systems. By including students—the future healthcare workforce—this research emphasises their role in shaping the future of nutrition care. While previous studies have identified gaps in nutrition education and training (Ní Chonaill et al. 2023; Keaver et al. 2018; Griffin et al. 2024; Khiri and Howells 2025; Krishnan et al. 2024; Thircuir et al. 2023), this study offers a unique contribution by examining the extent of nutrition integration in Ireland and its impact on healthcare practice.

## 2 Methods

### 2.1 Study Design Overview

This qualitative study explored the knowledge, practices and perceptions of HCPs and students regarding nutrition integration in Irish healthcare. Semi‐structured interviews addressed three research questions: (1) the level of nutritional knowledge of HCPs currently practicing; (2) perceived roles in nutrition integration; and (3) barriers to effective practice. Grounded in an interpretivist paradigm, the study aimed to co‐construct meaning from the experiences of HCPs and students, privileging multiple perspectives on nutrition integration.

A qualitative approach was chosen to capture the complexity of lived experience and to provide in‐depth insights into HCPs' perceptions and behaviours regarding nutrition integration, yielding patterns not accessible to quantitative methods (Tenny et al. 2022). Semi‐structured interviews balanced structure and flexibility, fostering both focused inquiry and the emergence of unanticipated insights (Ruslin et al. 2022; DeJonckheere and Vaughn 2019). Data were analysed using Braun and Clarke's reflexive thematic analysis (RTA), supporting systematic identification of patterns and themes while recognising participant subjectivity and researcher reflexivity (Braun and Clarke 2019, 2006; Byrne 2022).

### 2.2 Study Sampling and Recruitment

Medicine, nursing, pharmacy, physiotherapy and occupational therapy were selected as the healthcare professions of interest for their direct patient interactions and increased potential for lifestyle and health dialogues. Participants were recruited from primary care, community health and hospital settings to ensure diverse perspectives and experiences. Including students with clinical experience allowed for critical insight into their preparedness for clinical nutrition integration and the potential to shape future practice standards. Purposive and snowball sampling were used to achieve diverse representation across professional roles, experience levels and settings. Participants were recruited via professional networks and email invitation between December 2024 and April 2025. Inclusion criteria required direct involvement or experience in patient care relating to nutrition.

The study was granted ethical approval for the research by the ATU Research Ethics Committee after a full board review of the study proposal (REC‐ATUDG‐25‐0071). All participants were provided with an information sheet and gave informed, audio‐recorded consent. Interviews were recorded, transcribed verbatim and anonymised through the assignment of participant IDs and the removal of all identifiable information.

### 2.3 Data Collection

In‐depth, semi‐structured interviews were conducted online via Microsoft Teams or face‐to‐face, each lasting 20–60 min (mean = 29.25 min, SD = 10.54). All interviews were audio‐recorded with participant consent. The researchers (SOD, AG and SS) used an interview topic guide informed by key concepts from the literature, addressing nutrition knowledge, confidence in dietary counselling, previous training, integration barriers and referral pathways (see Table 1). The guide was piloted with two initial participants and deemed appropriate for subsequent interviews. Field notes were taken to document reflections, contextual details and potential biases, providing additional context to complement and enrich the data analysis.

**TABLE 1:** Semi‐constructive interview schedule guide questions and relevance.

Question	Relevance
Section 1. Knowledge of Nutrition	
What thoughts, feeling or perspective come to mind when you think about nutrition and your role?	Explores HCPs attitudes toward nutrition in healthcare
How would you describe your understanding of the role of nutrition in healthcare?	Assesses depth of understanding nutrition's role
Can you describe your level of knowledge regarding nutrition in patient care?	Measures self‐perceived nutrition knowledge
How confident do you feel about providing nutrition advice or guide in your practice/for your patient/s?	Identifies confidence in providing nutrition care
Do you see nutrition care as part of your role? Why or why not?	Assesses HCPs' views on nutrition integration
Have you received any form of nutrition education or training whether it be formal or informal?	Identifies training history and gaps
How adequate do you feel this training was or is in preparing you to address nutrition with patients?	Evaluates effectiveness of nutrition training.
Do you typically encounter or integrated nutrition into patient care? Can you provide any examples of situations where nutrition played a role?	Provides real‐world examples of nutrition integration.
Would you often discuss nutrition with your patients in your daily practice?	Measures frequency of nutrition discussions
How do you currently incorporate nutrition discussion into patient care?	Explores practical nutrition integration
Section 2. Nutrition Integration in Healthcare Practice	
How do you perceive the role of other healthcare professionals (e.g., dietitians, nurses, physiotherapists, occupational therapists, speech and language therapists) in supporting nutrition in health? Do they/should they have a role?	Assesses interdisciplinary understanding of nutrition and perceived roles
Tell me about a patient case where you needed help from a dietitian. What kind of help did you need?	Explores professional boundaries in nutrition
Do you perceive nutrition to be importance in the management of patient care? Can you give me any examples of where you've seen nutrition play a role in patient care?	Identifies perceived value of nutrition in care
In your view, what are the benefits of integrating nutrition into healthcare practices?	Highlights perceived advantages of nutrition integration
Section 3. Barriers to Nutrition Integration	
What are the main challenges in integrating nutrition care into your current role?	Identifies barriers to nutrition integration
Are there sufficient resources (e.g., nutritional guidelines, educational materials) available to you to provide to patients?	Measures resource availability for nutrition care.
Do you have access to nutrition specialists or dietitian if or when needed? If not, do you feel this has an impact on healthcare outcomes?	Assesses access to nutrition specialists
Are there any areas of nutrition you feel you lack knowledge or confidence in when providing advice to patients?	Identifies knowledge gaps and areas of confidence
What specific areas of nutrition do you believe you need more training in?	Identifies areas for further education
What limitations do you currently recognise in the **[X]** curriculum?	Examines gaps in formal education.
What are the current gaps in nutrition knowledge or skills in healthcare? Where do these gaps stem from and how might they be rectified?	Identifies and addresses knowledge gaps in healthcare.
Section 4. Support and Resources	
What would nutrition focus [substitute appropriate discipline] look like in an ideal world?	Explores ideal integration of nutrition in practice.
How could nutrition be better integrated into HCP education?	Identifies improvements for nutrition education.
Do you feel that there are sufficient opportunities for continuing education in nutrition within your professional environment?	Explores availability of ongoing nutrition education.
How do you think nutrition training for healthcare professionals could be improved or expanded in the future? Do you think it should be?	Gathers suggestions for improving nutrition education.
If you could suggest improvements to how nutrition is integrated into healthcare, what changes would you recommend?	Identifies recommendations for improving nutrition integration.
Is there anything else you would like to share about the role of nutrition in your practice or the challenges you face?	Provides an open‐ended opportunity for further insights.

### 2.4 Data Analysis

Interviews were transcribed and analysed using Braun and Clarke's six‐phase framework RTA analysis (Braun and Clarke 2019, 2006; Byrne 2022), chosen for its flexibility and iterative nature, which facilitated a thorough exploration of HCPs and students' perspectives. The six phases of RTA, as operationalised in this study, are summarised in Table 2.

**TABLE 2:** Reflexive thematic analysis process.

Step	Description
1. Data familiarisation	The researchers familiarised themselves with the data by transcribing the interviews, reading and re‐reading the transcripts and making initial notes.
2. Code generation	Initial codes were generated inductively using the comment feature in Microsoft Word to highlight relevant text excerpts for each code. Three transcripts were independently coded by A.G., S.S., L.R. and S.O'D., followed by consensus discussion to establish a unified coding framework. Throughout the analytic process, coding was conducted independently by A.G, S.S. and S.O'D., with regular reviews and discussions with the full research team to ensure reliability and minimise researcher bias.
3. Theme identification	Codes were collaboratively reviewed and refined by the research team, leading to the identification of overarching themes.
4. Theme review	Themes were iteratively reviewed and mapped to visually represent relationships and ensure coherence across the dataset.
5. Theme definition and naming	Themes were hierarchically organised, defined and related back to the research questions.
6. Report Production	Themes were contextualised in the final report, illustrated with participant quotations and reflexive notes to enrich interpretation and transparency.

### 2.5 Reflexivity Statement

The lead researchers, a postdoctoral researcher and two final‐year public health students with clinical and community experience, adopted a reflexive approach throughout the study. They regularly reflected on personal and professional perspectives, through field notes and ongoing discussions with the research team, to minimise bias and enhance transparency. This ensured diverse participant perspectives on nutrition integration were objectively represented in the analysis.

## 3 Results

### 3.1 Participant Characteristics

Thirty‐seven participants were interviewed, comprising qualified HCPs (*n* = 28) and students (*n* = 9) across medicine, nursing, pharmacy, physiotherapy and OT. As shown in Table 3, participants were predominantly female (84%) and included a broad range of clinical, primary care, community health and academic backgrounds. Qualified professionals included had between 1 and 30+ years clinical experience. Students were drawn from both undergraduate (pre‐registration with clinical experience) and postgraduate programmes. All of the postgraduate students had completed pre‐registration training and therefore had greater levels of clinical experience compared to the pre‐registration students included. Notably, one participant held dual roles as a physiotherapist and medical student; this individual is counted in both the ‘Healthcare Professionals’ and ‘Students’ categories (indicated by an asterisk*). Additionally, one general physician was also a qualified dietitian.

**TABLE 3:** Demographics characteristics of participants.

Demographic information	Healthcare professionals (*n* = 28)	Students (*n* = 9)
Gender	Male (2) Female: (26)	Male: (2) Female: (7)
Role/Field of Study	General Physician (6) Nurse (16) Physiotherapists (4)^*^ Pharmacy (2) Occupational Therapist (1)	Medicine (1)^*^ Nursing (3) Physiotherapy (3) Pharmacy (1) Occupation Therapy (1)
Years of experience	1–5 years (6) 6–10 years (5) 10–20 years (8) 20–30 years (2) 30+ years (7)	—
Specialisation	Primary Care (5) Orthopaedics (2) MSc Surgery (2) Rehabilitation (4) Paediatric (1) Public Health (1) Clinical Nurse Specialist (11) Pharmacy (2)	—
Work/Study setting	Primary Care (5) Hospital (17) Community (4) Self‐employed (2)	University—clinical placements (9)
Educational background	MB, BCh, BAO (6) BSc (7)^*^ MSc (1) MPharm (2) BNS (6) APRN (7)	Undergraduate (4) Postgraduate (5)^*^

Abbreviations: APRN, Advanced Practice Registered Nurse; BNS, Bachelor of Science in Nursing; MB, BCh, BAO, Bachelor of Medicine (MB) of Surgery (BCh) and of Obstetrics (BAO); MPharm, Master of Pharmacy.

^*^ One participant held dual roles as a physiotherapist and a medical student.

### 3.2 Thematic Findings

Through RTA, three overarching themes were developed, each with subthemes illustrating patterns in experiences and perspectives:

1. Professional boundaries, confidence and perceived competence in nutrition knowledge
2. Structural and logistical barriers to nutrition integration.
3. Variability in nutrition integration and its impact on healthcare practice.

Each theme is supported by participant quotes and reflexive commentary presented in Table 4, and is triangulated with insights from the initial coding and theme development processes.

**TABLE 4:** Developed themes, subthemes, sample codes, example quotes and reflexive notes.

Themes	Subthemes	Sample code		Example quote	Reflexive note
1. Professional Boundaries, Confidence & Perceived Competence in Nutrition Knowledge	1.1. Navigating Professional Boundaries & Scope of Practice	Professional boundaries & limitations in expertise	1a	‘I learned that it's a really specialised area, and that giving out information that I suppose I don't have qualifications under would be poor on my behalf’. (PS1)	Demonstrates strong awareness of professional limitations and ethical responsibility to remain within scope.
		Adherence to professional standards	1b	‘I do think it's not as easily said from the nurse because we are also responsible to a board and, you know, you have to be very careful what you say to people.’ (N1)	Highlights regulatory accountability as a factor shaping practice boundaries.
		Recognition of knowledge limitations	1c	‘You can't compete with someone who has done a four‐year degree in like nutrition that knows what they're on about.’ (OTS)	Reflects respect for dietetic expertise despite interdisciplinary role flexibility.
		Challenges in apply & communicating nutrition knowledge	1d	‘Sometimes it's not perceived well or taken well, whereas if that's coming from somebody whose job it is to talk about that, you know, in terms of a nutritionist or a dietician sometimes that then, well, you know, they're talking, like that's their specialist area and I'd probably more likely to take advice from them rather than, you know, this Doctor. And that is something that I certainly would have encountered’. (DS1)	Suggests credibility gaps hinder effective nutrition communication by non‐specialists.
	1.2. Confidence & Perceived Competence in Delivering Nutrition Advice	Limited confidence in nutrition knowledge	1e	‘I think what we have to offer as medical professionals; we just don't have that depth of knowledge for nutrition advice’. (GP1)	Reflects limited confidence due to gaps in formal education and training.
			1f	‘On a scale of 1–10, I would say 3–4 out of 10 when it comes to nutrition’. (N2)	Highlights self‐perceived inadequacy in nutrition competence.
		Personal interest influencing nutrition integration	1g	‘I think I would be confident enough in terms of talking about nutrition, but purely because I've had an interest in it’. (DS1)	Shows how individual motivation, rather than formal training, often drives nutrition competence, highlighting educational gaps in healthcare curricula.
			1h	‘I wouldn't, prior to my extracurriculars, have felt comfortable even mentioning anything beyond the loose sentences we were taught’. (PMS)	Reflects how confidence in nutrition practice is often dependent on person personal initiative, not professional education.
	1.3. Referral Pathways and Role Clarity	Referral for complex cases	1i	‘We would normally just refer on to the dietitian within work, if it was an issue that we weren't able to answer and it was an issue that we would have actually been impeding them, I suppose in there recovery’ (N1)	Emphasises reliance on specialists to mitigate risk of suboptimal care.
		Lack of established referral pathways in primary care	1j	‘There wasn't much [nutrition support] in primary care, and I think it was more isolated in that setting. But in the hospital…you would have actually have seen a dietitian quite regularly I suppose when you're working acutely, you're all very close together’. (OT1)	Illustrates disparity in access between acute and primary care settings.
		Role confusion	1k	‘Kind of knowing whether to refer to a dietitian or to a nutritionist, it's kind of sometimes a bit hard to distinguish between the two.’ (PS2)	Highlights a key barrier to interdisciplinary practice: confusion over professional roles undermines clarity in referral decisions…
2. Structural & Logistical Barrier to Nutrition Integration	2.1. Gaps in Nutrition Education & Training	Limited formal education in nutrition	2a	‘No, absolutely none. And I don't think we do either, from talking to my supervisor and placement, nutritional knowledge seems to be very limited in like occupational therapy’. (OTS)	Highlights the total absence of nutrition education in some professional curricula, pointing to systemic shortcomings in early training.
		Perceived training gap	2b	‘I think training for healthcare professionals is, I think there's a gap there certainly in my experience, there has definitely been a gap’. (GP1)	Confirms a widespread perception among HCPs of nutrition being marginalised in health education.
		Lack of CPD opportunities	2c	‘Certainty in my training days and ongoing, because we obviously do online or ongoing CPD all the time, but I have never done anything in nutrition, very little’. (GP1)	Illustrates a missed opportunity in professional development — an absence of structured, accessible CPD in nutrition.
	2.2. Training Deficits & Siloed Learning Environments	Desire for CPD & updated training	2d	‘I definitely think it would be valuable to have something on nutrition as part of CPD, even if it's just once a year. A focused CPD session on nutrition or dietary advice would be beneficial for physiotherapists, not just in terms of prescribing food but in providing general dietary advice that can assist in our practice’. (PS2)	Participant highlights a concrete, structured suggestion for professional development, rooted in practice relevance. Suggests growing demand among HCPs for discipline‐specific, applied training to improve patient care confidence and consistency.
		Lack of confidence due to outdated knowledge	2e	‘We're meeting patients every day that definitely we should be more empowered like to give information… we might feel more confident if you had maybe a course done in it every few years that you knew this is the new research and… you know, what the train of thought is now’. (N3)	Reflects the professional uncertainty stemming from outdated or inaccessible nutrition knowledge.
		Feelings of inadequacy	2f	‘I think we are probably failing our patients by not, I suppose, being adequately equipped with the skills to discuss it probably at length in depth’. (DS1)	Candid recognition of professional limitations; aligns with a call for reform in HCP preparation.
		Disciplinary silos in education	2g	‘I feel like they have just very much put barriers up that like you're doing nursing to become a nurse, you're doing dietetics or like nutrition to become that. They're not integrating it or seeing it as an umbrella that we could learn a few bits of things’. (N1)	Captures the compartmentalisation of healthcare education and the lack of integrative learning models. Suggests a missed opportunity for embedding nutrition across disciplines as a shared competency.
	2.3. Systemic Constraints in Nutrition Implementation	Resource scarcity in primary care	2i	‘Some people than just fall between two stools and they're just like, you know, there's just no resources and they say is there anyone I can speak to I really want to sort this out and there is nothing, there really is nothing, nothing to refer them, nowhere to refer them’. (N3)	Captures profound systemic absence of referral resources and pathways in community settings.
			2j	‘Out in the community we are in limbo land really and we just go with what we have’. (N3)	Highlights the isolation of community practitioners in the absence of structural support.
		Primary barriers to integration	2k	‘I suppose it's lack of education, lack of resources. You know, ideally you would have a dietitian see every patient, but it's just not feasible. So I would think, yeah, lack of resources and lack of time again’. (N2)	Recurring theme across disciplines – practical constraints are major barriers.
		Time constraints in education curriculums	2l	‘I think it's the timing and the demands of the course are already so extensive’. (PMS)	Suggests that integration of nutrition into education is challenged by curriculum overload.
		Reliance on primary care for nutrition support	2m	‘I think that very often a lot is kind of left up to the community or maybe up to primary care’. (DS1)	Highlights how nutrition care is frequently delegated to primary care, exposing gaps in system‐wide integration and shared responsibility.
			2n	‘You're probably fallen victim to like oh, I'm sure the GP or a public health nurse or someone would have picked it up before me, you know, which isn't always the case, like, because there's usually so many things going on that you're, it could just easily get missed’. (OT1)	Underscores the lack of clear accountability for nutrition, leading to missed opportunities for coordinated, integrated care.
		Disrupted referral continuity	2o	‘They were just welcoming a dietitian onto the team after not having one for months, which was absolutely bizarre given the amount of eating disorders they were seeing in the clinic and no dietitian to help on the team’. (OTS)	Shows how gaps in specialist availability disrupt continuity of care, especially for vulnerable groups, reflecting the need for stable, integrated dietetic support. Highlights the significant access barriers faced by patients due to a shortage of dietitians, delaying timely nutrition care and compromising outcomes.
		Prolonged waiting lists	2p	‘The waiting list for private referrals are so long because there are so limited dietitians, so it's a huge issue, it really is so huge. Last year, I tried to get two teenage girls referred to a dietitian, and I was told the waiting list was 6 to 9 months’. (N3)	
3. Variability in Nutrition Integration & its Impact on Healthcare Practice	3.1. Inconsistent Prioritisation	Nutrition de‐prioritised in complex cases	3a	‘I think when there's multiple things or more going on, nutrition seems to be pushed down the pecking order’. (PMS)	Demonstrates how competing clinical concerns can cause nutrition to be sidelined, even when relevant.
		Focus on medical treatment over nutrition	3b	‘A lot of my cohorts are like well, the most important thing is that we treat them medically and it's like it is important that they get the nutrition, but it's like more of a second‐hand thing that they're just like, Oh well, like we'll get to it eventually and it's not necessarily at the forefront, and it probably should be more at the forefront’. (DS2)	Highlights the entrenched medical model that prioritises pharmacological and procedural care over dietary interventions.
		Reliance on external resources	3c	‘Often we just have to use online resources and leaflets to give patients, printing them off and getting them there in the surgery which isn't really the ideal’. (N3)	Illustrates a lack of embedded, context‐specific nutrition support, replaced by generic tools.
		Inconsistent role in specialised vs. routine care	3d	‘We have improved in that since the Chronic Disease Management program came in general practice… that prompts us about nutrition, but prior to that, we probably weren't as good at checking people's BMI’. (GP1)	The lack of integration prior to the CDM program reinforces the idea that nutrition is often overlooked in routine care.
		Gaps in protocol awareness	3e	‘Even when it comes to diabetic patients, there are all these protocols as nurses we have for them for going home, which we wouldn't really be aware of to an extent because of all the renal diets, all these different types of diets’. (N1)	Underscores the gap between clinical guidelines and everyday practice, particularly around dietary complexity.
	3.2. Missed Opportunities & Consequences for Patient Outcomes	Delayed nutrition support in chronic disease management	3f	‘These people were, you know, like post‐transplant, some of them and like had come far in the system to being like rehab, like doing cardiac rehab class and they're coming that far and still didn't know stuff that I feel like they should have been told months ago in that process, you know?’. (PS3)	Illustrates missed opportunities for timely nutrition support along the care pathway.
		Role of nutrition in surgical recovery	3g	‘I think like, particularly in surgery, it's so important because no matter how well‐nourished you are leading up to it, well, patients usually aren't well nourished leading up to their surgery and then post‐surgery, they end up being in a deficit and they end up suffering consequences of it’. (DS2)	Highlights how insufficient perioperative nutrition can hinder recovery, underlining the value of structured nutrition support before and after surgery.
		Nutrition's role in physiotherapy practice	3h	‘One of the other physios had a girl in who was playing soccer and she's getting really injured and there was like I was in on the session… and the girl was like getting a lot of injuries in over 4 weeks and the physio did just ask her like what are you eating like how much would you say like talk me through a day of eating and she's like, yeah, I'm definitely not eating enough, but I just, I'm never hungry stuff like that’. (PS2)	Demonstrates how non‐dietetic staff can identify nutrition‐related red flags early, pointing to the need for basic nutrition skills across professions.
		Integrated care for eating disorders & mental health	3i	‘There should be a nutritionist when people are coming in with eating disorders or even like on the flip side like people coming in with weight issues and do you know, like impacting their mental health? And it's like if they fix the weight issues? Would they still be suffering with the mental health issues and low self‐esteem and like all this so’. (OTS)	Calls for integrated, interdisciplinary care; mental health and nutrition are interlinked but often treated in isolation.
		Uncertainty around best practices	3j	‘It's tricky because I feel like especially children with autism like you have food, food aversions and it's like, do you know a lot of them, like avoid fruit and vegetables and things of that and then there's research that's like, oh, we shouldn't push them to eat these certain types of foods. So I'd love to know kind of what, like, the kind of pathway for that is… just the best way to like approach that if that makes sense’. (OT1)	Highlights the uncertainty and lack of clear guidance faced by OTs when integrating nutrition care for patients with complex needs; need for clearer, evidence‐based nutrition guidance.
		Missed prevention opportunity in primary care	3k	‘We're in a very good place that we can intervene early and if we had the training and knowledge that we could maybe provide a lot of preventative healthcare in that area’. (GP1)	Reflects the untapped potential of primary care settings to deliver preventive dietary interventions if adequately resourced.
	3.3. Limited Integration in Team‐Based Nutrition Care	Need for cohesive interdisciplinary collaboration	3l	‘I think there's like different parts that are kind of pulled and like to form this jigsaw, you kind of need everyone like on the same hymn sheet’. (OTS)	Calls for alignment and consistency in interdisciplinary care approaches to integrate nutrition care optimally.
		Access difficulties for collaboration	3m	‘It just felt like it was very hard to like even to know where to find access to dietitians or nutritionists. Like you were just by your kind of isolated’. (OT1)	Indicates structural gaps that hinder effective referral and team‐based care.
		Working in parallel rather than together	3n	‘It just seems to be a lot of the time just working alongside each other rather than actually working together’. (OT1)	Exposes systemic disconnection between generalist HCPs and nutrition professionals, critiquing the absence of integrated communication pathways.
		Value of dietitians with MDT	3o	‘I really think I got like what my nutritionists tell me is gospel and I'm just like, the dietitians are vital to work with and like, I had a patient and he was like he was really ill anyway and then his bloods were getting worse and worse and worse and I couldn't figure out why and it turns out it was because he was starving and lacking in like the proper nutrition and the same with fluids and I wouldn't have been able to get that without nutrition support from dieticians’. (DS2)	Emphasises how collaborative nutrition support improves diagnostic accuracy and patient care.
		Knowledge‐sharing benefits	3p	‘I've often found we have really, really beneficial sessions where you know we have maybe the dietician sit down with us or nutritionist or somebody who's got a specialist interest in something and you then come away from those sessions with more knowledge, more empowerment and actually, you know, I suppose, get those messages across to your patients’. (DS1)	Reflects the positive impact of interdisciplinary sessions with nutrition specialists, enhancing knowledge and improving care through shared learning environments.

Abbreviations: DS, doctor/surgeon; GP, general physician; N, nurse; OT, occupational therapist; P, physiotherapist; PMS, physiotherapist and medical student; S, student.

#### 3.2.1 Theme 1: Professional Boundaries, Confidence and Perceived Competence in Nutrition Knowledge

This theme explores how HCPs' perceptions of their roles, boundaries and perceived competence influence when and how nutrition is integrated into practice, guiding decisions about when to advise, refer or communicate nutrition information.

##### 3.2.1.1 Navigating Professional Boundaries and Scope of Practice

Participants articulated a strong awareness of professional boundaries regarding nutrition care. They cited ethical and institutional messaging emphasising the importance of referring beyond their scope (Quotes 1a‐1b, Table 4). As one physiotherapy student explained:

As a student, we're being told if it's outside your scope, say it's outside your scope, because if you give advice, it's the wrong advice and there's repercussions on your head, and you shouldn't be doing that. (PS1)

Respect for the expertise of dietitians was widespread (Quote 1c, Table 4), with HCPs noting that patients were more responsive to advice from recognised specialists (Quotes 1d, Table 4).

They didn't want to hear so much from either the doctors that I shadow or me myself as a physiotherapist. (PMS)

##### 3.2.1.2 Confidence and Perceived Competence in Delivering Nutrition Advice

Confidence in delivering nutrition advice was highly variable and closely tied to the extent of formal training received (Quotes 1e‐1f, Table 4). Participants described limited nutrition education, resulting in hesitancy and uncertainty:

I always want to feel completely confident in things that I'm saying so when I don't have that…, it would be hard to kind of dive into conversation with patients. (PMS)

Where confidence was present, it was often attributed to personal interest or informal learning rather than formal instruction (Quotes 1g‐1h, Table 4):

Driven by personal interest really. (PS3)

Nutrition was also frequently regarded as common knowledge, rather than a formally taught skill:

I think it was more seen as common knowledge and you know that you're expected as a nurse… (N1)

##### 3.2.1.3 Referral Pathways and Role Clarity Within Professional Boundaries

Referral to dietitians was the main strategy for managing complex nutrition needs (Quote 1i, Table 4), with participants expressing caution about advising beyond their scope:

I'd probably just say look, there's a nutritionist that I can refer you on to that it's not my job to or like not my scope to be advising on that. (PS2)

Recognising personal limitations was seen as essential, particularly when nutrition became a barrier to patient progress:

I suppose more so in like referring on in that like it's so important to notice when a patient like when their nutrition is a barrier to their progression in physio. (PS3)

However, uncertainty about referral processes and role clarity was common, with concern raised about inadvertently giving unsolicited advice:

If you don't know that you should be referring on, you could just find yourself like unknowingly giving unsolicited advice. (PS2)

Access to dietetic support was more robust in acute settings, while primary care practitioners often faced isolation and unclear referral pathways (Quote 1j, Table 4). Role confusion persisted, with some unsure whether to refer to a dietitian or nutritionist (Quote 1k, Table 4) and others hesitant to consult colleagues due to workload pressures:

You just don't want to ask someone, unless it's like very necessary … you just know everyone's so swamped. (OT1)

#### 3.2.2 Theme 2: Structural and Logistical Barriers to Nutrition Integration

This theme illustrates how systemic gaps in education, resources and organisational support hinder the consistent and effective integration of nutrition care across healthcare settings.

##### 3.2.2.1 Gaps in Nutrition Education and Training

Formal nutrition training was limited across disciplines. The majority, if not all, expressed that nutrition was either limited or entirely absent from education curricula (Quotes 2a‐2b, Table 4). One physiotherapy student remarked:

We don't get taught anything about nutrition in our course. (PS1)

Likewise, opportunities for CPD in nutrition were scarce (Quote 2c, Table 4), as highlighted by participants:

I have never done anything in nutrition, very little. (GP1)

I haven't heard of any like nutrition courses offered like… even basic nutrition course. (OTS)

##### 3.2.2.2 Training Deficits and Siloed Learning Environments

Limited access to structured, profession‐specific CPD was a recurring concern. Participants advocated for regular, applied training, such as monthly or annual workshops, to build confidence and improve practice relevance (Quote 2d‐2e, Table 4). The need for early and continuous CPD was emphasised by participants.

More baseline knowledge should be provided from earlier on, more CPD workshops throughout teachings as well as training in the later years… to reinforce the role of nutrition and the fact that it is something that we should have more of a confidence in talking about with patients. (PMS).

HCPs described a lack of confidence due to outdated or insufficient knowledge, which limited their willingness to provide nutrition advice (Quote 2e, Table 4). Feelings of inadequacy in discussing nutrition with patients were also acknowledged (Quote 2f, Table 4).

The absence of interdisciplinary learning and collaboration reinforced professional silos and limited shared competence (Quote 2 g, Table 4). Additionally, the need for clear progression pathways in nutrition‐related competence was highlighted:

I think that the idea of having cleared like defined elevation or ascension pathways would be important. (P1)

##### 3.2.2.3 Systemic Constraints in Nutrition Implementation

Participants described widespread systemic barriers to delivering nutrition care, particularly in primary and community settings. Resource scarcity, limited staffing, and inconsistent referral pathways were commonly cited in relation to this (Quotes 2i‐2k, Table 4).

Some people… fall between two stools… there's nothing to refer them, nowhere to refer them. (PN)

I really wouldn't know who to contact who works in the community from that field. (PMS)

I think weight management is obviously a huge part of what we see in general practice and our resources again to deal with that are very poor. (GP1)

Time and capacity constraints within educational programmes (Quote 2l, Table 4) also limited exposure to nutrition content:

In a two‐year postgraduate course, there's already so much… I don't think there would even be time for a nutrition module. (PS1)

Responsibility for nutrition support was often deferred to primary care (Quotes 2m‐2n, Table 4), where professionals often felt underprepared and unable to provide consistent follow‐up. As one GP noted:

We certainly have a role, but we're not the best people to do it—we've neither the time nor the training. (GP1).

Continuity of care was further compromised by disrupted access to dietitians and long referral delays (Quote 2o‐2p, Table 4):

Traditionally our access to nutrition and dietetics has been so poor that I think we picked up that role. (GP1)

We would email community care and we would be waiting and waiting and waiting. (PN)

Participants also noted that publicly available nutrition resources were outdated and not credible to patients:

I feel like that's outdated information and also a bit like the patients themselves are probably looking at that they like, I know that's not the most updated information, come on, do you know? (PS3)

#### 3.2.3 Theme 3: Variability in Nutrition Integration and It's Impact on Healthcare Practice

This theme explores how differences in context, specialty and resources shape the prioritisation and delivery of nutrition care in everyday practice, highlighting both gaps and adaptive approaches that influence patient outcomes.

##### 3.2.3.1 Inconsistent Prioritisation of Nutrition

Participants described inconsistent prioritisation of nutrition across healthcare settings. In complex clinical environments, nutrition was frequently overshadowed by more immediate concerns or systemic pressures (Quote 3a‐3b, Table 4). One participant highlighted the practical difficulty of integrating nutrition advice into daily care:

You've so much to do that if they were to add in nutrition… if you were to go around to all your patients on a daily basis… a nutrition kind of advice session, you just don't have the time and that's the truth about it within your daily practice. (N2)

Inconsistencies were also evident at the individual clinician level, with providers emphasising nutrition or omitting it entirely:

I've seen situations where consultants will say, in a roundabout way, you know, the reason you're in hospital is because of your diet choices, and they will be quite firm with them, whereas other ones just won't say anything. (N2)

In primary care, participants relied on generic external resources due to the lack of embedded nutrition support (Quote 3c, Table 4).

I kind of just look for like resources online. (OT1)

In acute settings, prompts such as the Chronic Disease Management (CDM) programme aided integration, but awareness and adherence varied (Quote 3d‐3e, Table 4).

##### 3.2.3.2 Missed Opportunities and Consequences for Patient Outcomes

Delays or omissions in nutrition support were commonly reported across clinical areas. Missed opportunities were particularly noted in chronic disease management, with patients often reaching rehabilitation without prior nutrition intervention (Quote 3f, Table 4). Nurses highlighted that extended patient contact represented a missed opportunity, often due to unclear referral pathways:

We're obviously seeing that patient 12 hours a day, that's 39 hours a week…if you were able to provide some education to a degree, then they could go and find their own information… whether that be an outside private dietitian or private nutritionist. (N1)

Surgical care providers reported pre‐ and post‐operative nutritional deficits linked to inadequate nutrition support (Quote 3g, Table 4), while physiotherapists described unmanaged under‐eating in athletes due to referral challenges (Quote 3h, Table 4). Patient perceptions were also noted as a contributing factor to missed opportunities:

Also the patients themselves didn't fully perceive what the sort of necessity and importance that they should have. (PMS)

In mental health and eating disorder settings, nutrition was often siloed from psychological care (Quote 3i, Table 4). Similarly, a paediatric OT cited limited resources to address food aversions in autistic children (Quote 3j, Table 4), explaining:

You need like psychologists, you know, dietitians, SLTs and OTs coming together… and like, I've had to just say to parents, you know, we just don't have the resources here. (OT)

Primary care practitioners acknowledged the opportunity for early dietary intervention but reported lacking the training and structural support needed for meaningful impact (Quote 3k, Table 4).

##### 3.2.3.3 Limited Integration in Team‐Based Nutrition Care

Participants widely recognised the value of team‐based care in nutrition integration but described fragmented systems and inconsistent collaboration. One OT student compared navigating team‐based care to ‘assembling a jigsaw,’ emphasising challenges posed by poor communication and coordination among team members (Quote 3l, Table 4). Access to dietitians, especially in community settings, was limited, leaving professionals feeling isolated and unsure where to seek support (Quote 3m, Table 4).

Rather than coordinated teamwork, collaboration often occurred in parallel, with minimal integration across roles (Quote 3n, Table 4). Participants agreed that nutrition should be a shared responsibility. As one doctor remarked:

It shouldn't all be resting upon the dietitian to go through those things like we should be able to go through them as well. (DS1)

The importance of interdisciplinary care was reinforced by a medical student:

It's really [a] biopsychosocial model of patient care, and it feels a bit almost like counterproductive to ignore something that could play such a big impact. (PMS)

Dietitians were consistently valued within the IDT, with their input seen as essential to diagnostic clarity and patient outcomes (Quote 3o, Table 4). Participants also highlighted interdisciplinary learning sessions as beneficial for building confidence and capacity to provide basic nutrition support (Quote 3p, Table 4).

## 4 Discussion

The results of this study build on the findings from Eyemienbai et al. (2025), providing further comprehensive interdisciplinary accounts of how nutrition care is integrated into clinical practice among qualified HCPs and students in medicine, nursing, pharmacy, physiotherapy and OT. While participants consistently recognised nutrition as a core aspect of professional responsibility, echoing global initiatives (e.g., United Nations Decade of Action on Nutrition) and previous literature (Thow et al. 2025; Keaver et al. 2022), the translation of this recognition into routine clinical practice remains constrained by entrenched professional, educational and systemic barriers. This aligns with global evidence that, despite HCPs being well‐positioned to deliver foundational nutrition support due to frequent patient contact (Agwara et al. 2024; McClinchy et al. 2015; Ball et al. 2010, 2014; Aggarwal et al. 2020; Crowley et al. 2019), significant deficits in training, system structure and professional boundaries continue to constrain effective integration.

### 4.1 Interpretations of Findings

#### 4.1.1 Professional Boundaries, Confidence and Perceived Competence

All participant groups recognised the importance of providing first‐line evidence‐based nutrition advice and facilitating referrals as part of their professional role (Ní Chonaill et al. 2023; Ball et al. 2010; Wong et al. 2025; Thow et al. 2025; Keaver et al. 2022). However, this ideal is consistently undermined by ambiguous role boundaries, insufficient training and entrenched systemic barriers (Keaver et al. 2018; Agwara et al. 2024; Wong et al. 2025). Echoing previous studies, nutrition care in Ireland remains largely reactive, not proactively embedded within patient management (Keaver et al. 2018). Notably, many HCPs, particularly nurses, perceived nutrition as ‘assumed knowledge’ rather than a formally taught or institutionally supported skill. This disconnect leaves HCPs lacking confidence, increases risks to patient safety and contributes to unmet patient expectations, as limited nutrition education may result in inconsistent competence and delivery of evidence‐based care (Ní Chonaill et al. 2023; McMonagle et al. 2023; Griffin et al. 2024; Keaver et al. 2021). There is strong evidence that targeted, competency‐based nutrition education is critical to building HCPs' self‐efficacy and readiness to deliver effective nutrition care (Griffin et al. 2024; Mogre et al. 2016). For meaningful improvements, education must go beyond foundational knowledge to ensure HCPs develop practical skills and confidence in addressing dietary risk factors across diverse clinical settings (Griffin et al. 2024; Livne 2019).

Where confidence existed, it stemmed largely from personal initiative rather than structured education, a finding with significant implications for the consistency and reliability of advice provided (Carter et al. 2022). This reliance on self‐directed learning results in inconsistent care, with practice shaped more by individual interests than by standardised, evidence‐based guidelines. Similar findings were reported in 2018 by Chapple et al. (2018), who observed that in traumatic brain injury care, HCPs' attitudes and beliefs often drive nutrition practice more than formal guidance, highlighting the need for improved interdisciplinary education to ensure consistency and effectiveness in nutrition care. Even among those with a strong personal interest in nutrition, participants widely acknowledged the limits of their expertise and identified referral to specialists as a central aspect of their role in providing nutrition care (Lepre et al. 2023). An Australian study investigating a systemised, interdisciplinary approach to malnutrition care reported improved nutrition care processes and enhanced coordination among HCPs, which resulted in better patient reported experiences and highlighted the value of interdisciplinary collaboration in improving overall outcomes (Bell et al. 2021).

Professional organisations messaging emphasising strict adherence to professional boundaries, instructing students to ‘stay in your lane’ further curtailed nutrition engagement, reinforcing reliance on dietitians and limiting true interdisciplinary collaboration (Agwara et al. 2024; Wong et al. 2025). This study also challenges assumptions that all medical HCPs are viewed as credible sources of nutrition advice (Keaver et al. 2018; Ball et al. 2014, 2015). Doctors and physiotherapists often reported that patients preferred advice from dietitians, and participants perceived doctors' primary responsibility as facilitating referrals rather than providing direct advice (Griffin et al. 2024; Lepre et al. 2023; Caldow et al. 2022). These HCPs are on the frontline of patient care and ideally placed to offer first‐line dietary advice; however, without adequate knowledge, they may feel ill‐equipped, leading to missed opportunities for early intervention and consistent advice (Keaver et al. 2018; Caldow et al. 2022). Upskilling HCPs to competently identify nutrition‐related problems, provide first‐line evidence‐based advice for general nutrition queries, and refer appropriately through clear pathways could optimise patient care, while enabling dietitians to focus on more specialist patient cases with complex nutrition problems.

Participants described widespread uncertainty regarding referral pathways, especially when distinguishing between dietitians and nutritionists, and expressed concerns about burdening colleagues, a barrier particularly prominent in primary care. Given the difference in regulation, qualifications and training, it is essential for all HCPs to understand the scope of practice of registered dietitians, registered nutritionists and nutritional therapists (AfN 2018). Consistent with prior research, factors such as limited access to dietitians, patient motivation and financial constraints further restricted effective referral and nutrition care (Lepre et al. 2023). These findings collectively underscore the need for clearer interprofessional boundaries and standardised, accessible referral frameworks across the healthcare system (Carter et al. 2022; McClinchy et al. 2015; Wong et al. 2025; Kelly et al. 2022).

#### 4.1.2 Structural and Logistical Barriers to Nutrition Integration

Nutrition education remains systematically underrepresented in both undergraduate pre‐registrationand postgraduate curricula, echoing longstanding concerns from research across Europe and Australia (Ní Chonaill et al. 2023; McMonagle et al. 2023; Griffin et al. 2024; Crowley et al. 2019; Keaver et al. 2021). This gap is exacerbated by policy frameworks, such as those of the World Federation for Medical Education (2020), which do not require nutrition competencies for medical graduates (Keaver et al. 2022; Schwill et al. 2022). Participants in this study repeatedly identified the absence of structured nutrition education and the scarcity of relevant CPD opportunities as key obstacles to clinical competence, a finding corroborated by recent systematic reviews showing that inadequate nutrition training undermines knowledge, skills and confidence to deliver effective nutrition care (Crowley et al. 2019). Where available, CPD was often described as poorly tailored, inaccessible due to time constraints, or insufficiently aligned with daily practice. This presents an opportunity for CPD providers such as the Irish Nutrition and Dietetic Institute (INDI) to collaborate with the Department of Health to signpost HCPs to readily‐available, evidence‐based nutrition CPD learning, and develop tailored resources to support HCPs in delivering first‐line dietary advice.

A striking reality is that most HCPs receive less than two hours of nutrition education during formal training (Khiri and Howells 2025), a consequence of curriculum crowding, limited advocacy, and a shortage of specialist educators (Kris‐Etherton et al. 2014; Crowley et al. 2019). Participants reported a lack of confidence in discussing nutrition with patients. The use of patient simulations in HCP education could better prepare HCPs to address nutrition and nutrition‐related topics, such as weight management, with evidence of success seen in dietetics education in improving communication skills and confidence (Buchholz et al. 2020). Participants advocated for accessible CPD that is both discipline‐specific and interprofessional in nature, with online modules highlighted as a scalable solution for enhancing interprofessional competence, particularly in community settings (Hicks‐Roof 2022). While discipline‐specific training supports role clarity and depth of knowledge, interprofessional learning was seen as complementary in fostering collaboration and a shared understanding of nutrition care across healthcare teams. Consistent with the literature, these findings reinforce that while comprehensive nutrition expertise is not universally expected, all HCPs require a clear understanding of allied HCP roles and robust referral skills to dietitians, ensuring timely specialist input when necessary (Caldow et al. 2022).

#### 4.1.3 Variability in Nutrition Integration and Its Impact on Healthcare Practice

Despite widespread acknowledgment of the value of interdisciplinary collaboration, access to dietitians and referral pathways was often described as fragmented, particularly in primary and community care (Carter et al. 2022; McClinchy et al. 2015; Kelly et al. 2022). The participants described professional isolation, with frequent reports of patients ‘falling between stools’ due to delays or inability to access dietetic support. These structural barriers translated into inconsistent delivery, with nutrition care often deprioritised amidst competing clinical demands, especially in acute, surgical and complex community settings. A 2021 study on older adults' experience of malnutrition in Ireland reported the consequences of fragmented interdisciplinary collaboration and lack of dietetic service availability, with patients relying heavily on informal carers and highlighted the need for improved interdisciplinary communication and better training for healthcare professionals (Reynolds et al. 2021).

Practice nurses and OTs highlighted the challenge of coordinating interdisciplinary care for complex cases such as paediatrics and mental health. GPs often relied on generic handouts or external referrals hampered by lengthy waitlists. This resulted in HCPs working in silos, providing ‘parallel’ rather than integrated care (Marshall et al. 2019; Tappenden et al. 2013). While participants valued opportunities for collaborative learning, true integration remained rare, consistent with literature showing that interdisciplinary education, such as dietetic students coaching medical students, enhances interprofessional skills and patient care (Crowley et al. 2019).

Collectively, these findings highlight a pressing need for reform across education, training and service delivery to close the persistent gap between recognition and implementation of effective nutrition care.

### 4.2 Implications

These findings have important implications for policy, education, and clinical practice. There is a clear need for:

- Curriculum reform: Integration of mandatory, interdisciplinary, first‐line evidence‐based nutrition content at both pre‐registration and postgraduate levels;
- Accessible, protected CPD: Expansion of online, role‐appropriate CPD modules to foster interprofessional learning and competence;
- Standardised referral frameworks: Development of national guidelines, clear interprofessional boundaries, and interdisciplinary frameworks to streamline nutrition care and escalation pathways;
- Practice‐based learning: Greater use of simulations and case‐based learning to model interdisciplinary collaboration and enhance referral skills;
- System‐level change: Embedding dietitians within primary and community care teams and ensuring timely access for all patient populations.

### 4.3 Strengths and Limitations

This study offers a timely and highly relevant exploration of an under‐researched topic, contributing valuable insights with potential implications for practice, education and policy. The inclusion of participants from diverse professional backgrounds provided a rich, multifaceted perspective on nutrition integration in healthcare. As a qualitative study, no measure of competence was captured and findings are grounded in participants' subjective experiences and interpretations, which may be influenced by social desirability or researcher perspective. While robust reflexive analysis and triangulation with field notes enhanced the credibility and trustworthiness of the results, the absence of patient perspectives limits the scope of insights presented.

Additionally, the low number of male participants, alongside the predominance of female voices, may have restricted the diversity of views represented. The sample may also reflect a degree of self‐selection bias, as two of the 28 healthcare professionals had previously completed a pre‐registration healthcare qualification, potentially indicating a cohort with a greater prior interest in nutrition than a more representative sample. Furthermore, while some participants reported engaging in informal nutrition learning, the nature and quality of this learning were not clearly defined; given that informal sources are not always evidence‐based, this represents an additional limitation. Inclusion of these perspectives in future research would offer a more comprehensive understanding of nutrition integration in healthcare practice.

### 4.4 Recommendations for Future Research

Future studies should employ longitudinal and mixed‐methods designs to assess the impact of educational reform and care models on nutrition practice and outcomes. A mixed‐methods approach could combine quantitative data, such as knowledge and competency assessments, referral rates, patient nutritional status and clinical outcomes, with qualitative data exploring HCP and patient experiences. Including patient perspectives is essential to capture lived experiences and expectations, providing a complete understanding of system‐wide barriers and opportunities.

Areas warranting further investigation include: the impact of mandatory, interdisciplinary, first‐line nutrition content at pre‐registration and postgraduate levels; the quality and nature of informal learning utilised by HCPs and its role in shaping nutrition competence; the outcomes of standardised referral frameworks and national guidelines on timely patient care; the use of simulations and case‐based learning to improve HCP confidence, communication and referral skills; and the benefits of embedding dietitians within primary and community care teams to enhance patient access and system integration. Addressing these gaps would generate actionable insights to guide curriculum development, workforce planning and policy initiatives in nutrition care.

## 5 Conclusion

This study highlights a persistent gap between recognition and practice of nutrition care rooted in systemic, educational and cultural factors. Addressing these challenges through targeted education reform, clear referral pathways and stronger interdisciplinary collaboration is essential for improving patient outcomes and reducing the burden of nutrition‐related disease in Ireland. Enhancing nutrition education and strengthening system‐level support for HCPs and students will improve their confidence, competence and delivery of nutrition care in clinical practice, helping to address missed opportunities in prevention and patient management.

## Author Contributions

All authors conceptualised the study and supported the development of the overall research plan. S.O., A.G. and S.S. completed data collection, data extraction and synthesis and drafted the manuscript. L.R. supported data extraction and evidence synthesis and was involved in manuscript preparation. G.M., L.K. and R.D. supported evidence synthesis and were involved in manuscript preparation and refinement. All authors have read and approved the final manuscript.

## Funding

The research leading to these results has received funding from the Nutrition Education for HealthCare Professionals (NEHCP) project co‐funded by the Erasmus+ Programme of the European Union under the Grant agreement number 2024‐1‐IE02‐KA220‐HED‐000244217.

## Disclosure

This study was conducted according to the guidelines laid down in the Declaration of Helsinki and all procedures involving research study participants were approved by Atlantic Technological University, Galway, Research Ethics Committee (REC‐ATUDG‐25‐0071). Verbal informed consent was obtained from all subjects. Verbal consent was witnessed and formally recorded.

## Conflicts of Interest

The authors declare no conflicts of interest.

## Data Availability

The data that support the findings of this study are available from the corresponding author upon reasonable request.
